# Outcomes of Heart Transplantation in Single-Ventricle Physiology: A Retrospective Single-Center Experience with Emphasis on Surgical Complexity

**DOI:** 10.3390/jcm15051714

**Published:** 2026-02-24

**Authors:** Szymon Pawlak, Joanna Śliwka, Roman Przybylski, Agnieszka Kuczaj, Małgorzata Szkutnik, Piotr Przybyłowski, Tomasz Hrapkowicz

**Affiliations:** 1 Department of Cardiac, Vascular and Endovascular Surgery and Transplantology, Silesian Center for Heart Diseases, Medical University of Silesia, 41-800 Zabrze, Poland; s.pawlak@sccs.pl (S.P.); j.sliwka@sccs.pl (J.Ś.); p.przybylowski@sccs.pl (P.P.); t.hrapkowicz@sccs.pl (T.H.); 2 Department of Cardiac Transplantation and Mechanical Circulatory Support, Wroclaw Medical University, 50-556 Wrocław, Poland; roman.przybylski@umw.edu.pl; 3 Department of Pediatric Cardiology and Congenital Heart Defects, Faculty of Medical Sciences in Zabrze, Silesian Center for Heart Diseases, Medical University of Silesia, 41-800 Zabrze, Poland; m.szkutnik@sccs.pl

**Keywords:** heart transplantation, single-ventricle heart physiology, Fontan procedure

## Abstract

**Background:** Patients with single-ventricle physiology represent a high-risk group for heart transplantation. Due to complex anatomical and physiological challenges, including multiple prior sternotomies, pulmonary artery abnormalities, and systemic consequences of altered circulation, they represent both a surgical and a clinical challenge. We aimed to analyze perioperative challenges, as well as early and long-term complications, in this specific group of patients. **Methods:** We performed a retrospective data analysis of a high-volume heart transplant center, focusing on patients with single-ventricle physiology who were scheduled for heart transplantation due to end-stage heart failure. We retrospectively analyzed the period from the beginning of the transplant program in November 1985 to the end of November 2024. **Results:** Among 1553 transplanted patients (adults and children), 29 were transplanted due to congenital heart disease (congenital valvular disease not included). In this group, nine patients were transplanted due to end-stage heart failure in the course of single-ventricle physiology. Age at transplantation ranged from 7 to 31 years (median, 17 years), and body weight ranged from 15 to 69 kg (median, 47.9 kg). All nine patients referred for heart transplantation presented with single-ventricle physiology. Their underlying congenital heart defects were heterogeneous and included hypoplastic left heart syndrome (HLHS), double-outlet left ventricle (DOLV), transposition of the great arteries (TGA) with associated ventricular septal defects (VSDs), atrial septal defects (ASDs), valvular abnormalities such as tricuspid and or pulmonary valve atresia or stenosis, systemic or atrioventricular valve regurgitation, and vascular abnormalities, including right-sided aortic arch, aortic coarctation, and pulmonary artery hypoplasia, stenosis, or occlusion, as well as associated pulmonary vascular abnormalities such as left pulmonary artery stenosis and MAPCAs. All patients had previously undergone staged palliative procedures, including Norwood, Hemi-Fontan, Fontan, bidirectional Glenn, modified Blalock–Taussig shunts, Bjork–Fontan, or pulmonary artery banding, often with repeated interventions such as balloon angioplasty, stent placement, or MAPCA closure. Extracardiac comorbidities were common and included coagulopathies, protein-losing enteropathy, hepatic dysfunction, and chronic venous insufficiency. Preoperative functional status was markedly impaired in all patients (NYHA III-IV, INTERMACS 3-4), with severely reduced exercise capacity and thrombotic events in several individuals. Perioperative transplant surgical strategies included femoral cannulation in four cases and standard aortic and caval cannulation in five cases. Pulmonary artery reconstruction was required in all patients. Extended donor pulmonary arteries were applied in eight cases, while a bifurcated Dacron prosthesis was utilized in one patient. Perioperative mortality was 33%, with three deaths attributed to bleeding and hemodynamic instability, while overall mortality was 44% including one late death unrelated to transplantation. Protein-losing enteropathy, although persistent in the immediate postoperative period, resolved in all surviving patients, underscoring the transformative impact of transplantation. **Conclusions**: These findings emphasize the importance of individualized surgical planning, extended donor pulmonary artery harvesting, and careful preoperative coordination. Heart transplantation remains a viable and life-extending option for selected single-ventricle patients, despite the significant technical and clinical challenges involved.

## 1. Introduction

The increasing survival rates of children born with single-ventricle physiology, largely due to advancements in staged surgical palliation, have led to a growing population of patients reaching adolescence and adulthood with complex congenital heart disease [1,2]. Procedures such as the Fontan and bidirectional Glenn have enabled these patients to achieve better outcomes; however, the long-term viability of these palliative strategies is often limited [3,4,5]. Many patients ultimately experience progressive heart failure, necessitating orthotopic heart transplantation (OHT) as a final therapeutic option [2,6].

Single-ventricle physiology encompasses a heterogeneous group of complex congenital malformations. The most frequently encountered underlying diagnoses include hypoplastic left heart syndrome (HLHS), tricuspid atresia, double-inlet left ventricle, and unbalanced atrioventricular septal defect, with less common contributions from complex double-outlet right ventricle variants with ventricular hypoplasia [7,8]. Across surgical and heart failure cohorts, HLHS represents approximately 30–40% of patients with single-ventricle physiology surviving beyond infancy, while tricuspid atresia and double-inlet ventricle together account for an additional 20–30%, and unbalanced atrioventricular septal defect comprises most of the remaining cases [7,8,9]. Contemporary management of single-ventricle defects relies on staged surgical palliation, culminating in the Fontan circulation, which directs systemic venous return passively to the pulmonary arteries in the absence of a subpulmonary ventricle [7,10].

Advanced heart failure in congenital heart disease (CHD) is defined by refractory symptoms, impaired functional status, and evidence of systemic and pulmonary circulatory compromise despite optimal medical and interventional therapy. This condition often leads to clinical deterioration that ultimately necessitates consideration of OHT or advanced mechanical support. Importantly, data from contemporary cohorts indicate that the onset of advanced heart failure (HF) in adult congenital heart disease patients is associated with a substantially increased risk of death, highlighting the severe clinical burden in this population [11]. A fundamental determinant of poor outcomes in congenital heart disease with HF is the heterogeneity and complexity of underlying cardiac defects.

Many adults with CHD have either functionally single-ventricle physiology, for example after Fontan palliation, or systemic right ventricles, anomalies known to predispose to progressive ventricular dysfunction. These circulatory patterns differ from the typical left ventricular dysfunction seen in other etiologies of HF, presenting unique biomechanical and hemodynamic stresses that accelerate myocardial deterioration. Additionally, the systemic right ventricle lacks the morphological architecture to support systemic pressures long term and, over time, demonstrates progressive adverse remodeling, diastolic and systolic dysfunction, and arrhythmogenesis. This predisposition to failing ventricular performance is also exaggerated by prior surgical scars, residual lesions, and patch material that can alter myocardial compliance and conduction pathways [12]. While staged palliation and the Fontan procedure have significantly improved childhood survival, they establish a circulation characterized by chronically elevated central venous pressure, limited preload reserve, and reduced cardiac output, even in clinically stable patients [10,13]. Over time, the Fontan circulation predisposes patients to a constellation of complications collectively referred to as Fontan failure, which may occur despite preserved ventricular systolic function [10,13,14]. Long-term follow-up studies indicate that 20–40% of Fontan patients develop clinically significant Fontan failure by early adulthood, manifesting as exercise intolerance, refractory fluid retention, protein-losing enteropathy, plastic bronchitis, or progressive ventricular dysfunction [13,14,15].

In single-ventricle patients, pulmonary vasculature and pulmonary hypertension are often difficult to assess and may further complicate transplant candidacy or lead to the selection of oversized donor hearts [16]. In patients with a Fontan circulation, systemic venous return is directed passively into the pulmonary arteries through a total cavopulmonary connection, resulting in a nonpulsatile, low-pressure pulmonary circuit in the absence of a subpulmonary ventricle. In this physiology, pulmonary blood flow is principally governed by central venous pressure and pulmonary vascular resistance, and mean pulmonary artery pressure seldom reaches thresholds used to define pulmonary arterial hypertension in biventricular circulations. Consequently, even modest increases in pulmonary vascular resistance have a disproportionate impact on transpulmonary flow and systemic cardiac output, as the Fontan circulation is inherently preload-dependent and lacks the ability to augment transpulmonary gradients via ventricular work [17]. Accurate invasive assessment of pulmonary hemodynamics in Fontan patients is challenging. In the absence of a subpulmonary ventricle, conventional pulmonary arterial pressure measurements are often deceptively low and may not reliably reflect clinically relevant pulmonary vascular obstruction or elevated resistance. Furthermore, altered anatomy, surgically created conduits, and collateral flow complicate hemodynamic interpretation. Accordingly, pulmonary vascular resistance is typically calculated indirectly, and pressure measurements must be integrated with advanced imaging modalities, including transthoracic echocardiography, cardiac magnetic resonance imaging, and computed tomography, to comprehensively characterize pulmonary vascular structure and flow [18].

Additionally, among Fontan patients, chronically elevated lymphatic pressures are believed to promote abnormal lymphatic drainage into the bronchial tree and the formation of proteinaceous airway casts, causing airway obstruction and respiratory distress. This rare lymphatic-related complication, known as plastic bronchitis, may further complicate outcomes in these patients [19].

A hallmark of the CHD population is an extensive surgical history. The vast majority of CHD patients have undergone multiple cardiac surgeries during childhood to palliate or correct structural defects, frequently involving sternotomies, vessel reconstructions, and patch placements. These prior procedures contribute to complex intraoperative anatomy that increases the technical difficulty of transplant surgery, prolongs ischemic times, and predisposes patients to bleeding complications. Extended operative times and reconstruction requirements correlate with higher perioperative morbidity and mortality [16]. Furthermore, after multiple surgical interventions patients may exhibit increased alloimmune sensitization due to exposure to blood products and homograft materials, resulting in elevated panel reactive antibody (PRA) levels that challenge donor matching and increase the risk of acute and chronic rejection after transplantation [20]. According to the 2025 International Society for Heart and Lung Transplantation (ISHLT) Registry, adult patients with congenital heart disease exhibit the highest risk of one-year post-transplant mortality, comparable to that observed in patients requiring mechanical ventilation at the time of transplantation [21].

This study investigates the outcomes of heart transplantation in patients with predominantly single-ventricle physiology. By examining perioperative challenges, survival rates, and postoperative complications, we aim to provide insights into optimizing the care pathway for this high-risk patient group [22].

## 2. Materials and Methods

We retrospectively analyzed the medical records of a high-volume heart transplant center to identify single-ventricle patients who underwent heart transplantation. The data analysis covered the period from 5 November 1985, to the end of November 2024.

Key metrics included patient demographics, anthropometric data, surgical procedures performed up to the time of transplantation, perioperative complications, and survival outcomes. Additional data, such as waiting time on the transplant list, were also recorded and analyzed. All patients were managed at a single medical center by a multidisciplinary team specializing in congenital heart disease and transplant care.

The study was conducted as a retrospective, observational analysis and did not meet the criteria for a medical experiment. As all data were fully anonymized, precluding the identification of individual patients, the local bioethics committee determined that the requirement to obtain informed consent did not apply.

Given the small cohort of patients with single-ventricle physiology, analyses were primarily descriptive. Continuous variables are reported as medians with interquartile ranges (IQR), minimum and maximum values, or as means ± standard deviation (SD), depending on distribution. Categorical variables are presented as counts and percentages. Perioperative and long-term outcomes, including survival and postoperative complications, were summarized without formal hypothesis testing or multivariable modeling. All calculations and tabulations were conducted using established statistical approaches suitable for retrospective observational data, with attention to accuracy in small-sample analyses.

## 3. Results

**Figure 1:**
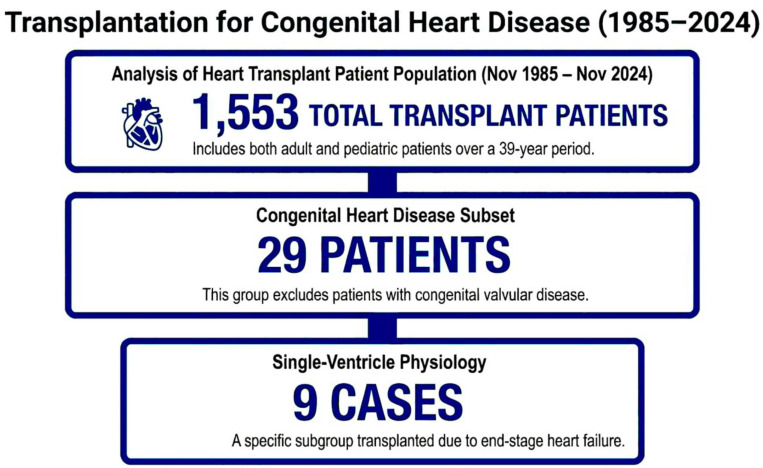
Flowchart of the study.

Among 1553 patients (adults and children) transplanted during the period from 5 November 1985 to the end of November 2024, 29 patients were transplanted due to congenital heart disease (congenital valvular disease not included). In this subgroup, nine patients were transplanted due to end-stage heart failure in the course of single-ventricle physiology. The flowchart of the study is presented in Figure 1.

The median age at transplantation was 17 years (IQR, 11.5–22; mean, 17.3 years; SD, 8.25). Three patients were grown-up congenital heart disease patients (over 18 years of age), and five patients were female. Among them, seven patients underwent heart transplantation following the Fontan procedure, and two following the bidirectional Glenn procedure. Patients had a median body weight of 54 kg (IQR, 30–63; mean, 49.2 kg; SD, 21.0). The average waiting time on the transplant list was 162.6 days (SD, 156.7), with a median of 95 days (IQR, 27.5–307 days). Seven patients had previously undergone three sternotomies as part of staged palliation, while the remaining two patients had undergone two prior sternotomies. Baseline patient characteristics are presented in Table 1.

### 3.1. Perioperative Management

The patients underwent extensive preoperative evaluation, including laboratory tests, right heart catheterization, and diagnostic imaging, such as echocardiography, chest computed tomography, and cardiac magnetic resonance imaging, when feasible. Perioperative management involved ensuring the availability, at the start of the procedure, of higher-than-standard quantities of red blood cell concentrates, platelet concentrates, fresh frozen plasma, and clotting factors to provide adequate hemostatic support.

### 3.2. Transplant Procedure

During the transplantation procedure, femoral cannulation for cardiopulmonary bypass was employed in four patients, as this approach provided safer resternotomy in cases where adhesions were particularly severe. In the remaining five patients, standard cannulation of the aorta and the superior and inferior venae cavae was successfully performed. Establishing extracorporeal circulation with standard cannulation in these cases required meticulous isolation of the great vessels.

The native pulmonary arteries of the patients had been remodeled by previous palliative surgical procedures. To reconstruct the pulmonary vessels, donor hearts were harvested with both pulmonary arteries preserved in eight patients. In one patient, a bifurcated Dacron prosthesis was utilized to reconstruct the pulmonary artery. Harvesting of the donor pulmonary arteries was not possible due to multiorgan procurement, including the lungs.

### 3.3. Immunosuppressive Regimen

Recipient sensitization was assessed at the time of listing using panel reactive antibody (PRA) testing. PRA levels were determined by the complement-dependent cytotoxicity (CDC) assay. A PRA value greater than 10%, in accordance with the standards of the local transfusion medicine laboratories performing the tests, was considered indicative of clinically relevant sensitization to donor antigens. Donor–recipient crossmatching was performed in all cases. Crossmatching was routinely conducted retrospectively; however, in sensitized recipients and at the discretion of the operating surgeon, a prospective crossmatch was performed, and heart transplantation was undertaken only after exclusion of a positive result. Induction immunosuppressive therapy with basiliximab was administered in two recipients. All patients received maintenance immunosuppression according to the institution’s current protocol, consisting of a calcineurin inhibitor, tacrolimus or cyclosporine, mycophenolate sodium, and high-dose methylprednisolone with gradual tapering, followed by prednisone with dose reduction over the first post-transplant year.

### 3.4. Perioperative and Early Mortality

Three patients, representing 33% of the cohort, died during the early perioperative and postoperative period. One patient died on the first postoperative day following transplantation due to excessive, uncontrollable bleeding. Two patients died during the initial postoperative hospitalization: one patient on postoperative day 65 due to multiorgan failure, the patient after pulmonary artery reconstruction with a Dacron graft, and the second on postoperative day 168 due to pulmonary cytomegalovirus infection.

### 3.5. Long-Term Follow-Up

Among the patients who survived the early postoperative period, four males and two females, complications related to Fontan circulation and the Glenn procedure resolved completely. Within the first year, there were no signs of liver failure, clotting disorders, or protein-losing enteropathy. One female patient, aged 21 years, died on postoperative day 353 due to surgical complications following a gynecological procedure, specifically bleeding and hemorrhagic shock after ovarian cyst excision. The remaining five patients are alive, with a median observation time of 154 months (IQR, 97–154 months). The median age of the survivors at follow-up was 27.7 years (IQR, 22.5–30.6 years).

Among major transplant-related complications, three patients developed graft vasculopathy, with one patient undergoing multiple percutaneous transluminal coronary angioplasty procedures. In one patient, balloon angioplasty of a stenosis at the anastomotic site between the neoaorta and the descending aorta was performed two years after transplantation. Two patients had arterial hypertension. None of the patients had diabetes or chronic kidney disease of grade 3b or higher, according to the KDIGO classification. All patients exhibited normal graft systolic and diastolic function, with no valve abnormalities.

## 4. Discussion

The outcomes observed in this study highlight the unique challenges and complexities associated with heart transplantation in single-ventricle patients. Consistent with findings from other authors reporting outcomes of heart transplantation in patients with single-ventricle physiology [23], our results demonstrated a high first-year mortality. However, long-term survival, conditional on surviving the first year, appears to be acceptable or even promising. One of the most significant findings is the necessity for individualized surgical planning for each patient. The timing of transplantation in patients with single-ventricle physiology is particularly difficult to determine. Data from recent ISHLT registry slides indicate that patients with congenital heart disease face very high mortality after heart transplantation [21].

Determining the optimal therapeutic window for transplantation is challenging and requires a multidimensional assessment, including evaluation of ventricular function, end-organ status, vascular access, and ethical considerations. Ideally, transplantation would be considered in patients with preserved ventricular function, stable end-organ performance, and adequate vascular access, before the development of irreversible multi-organ dysfunction. Conversely, exposing a patient who has undergone multiple prior cardiac surgeries and catheter-based interventions, yet remains clinically stable, to a major procedure associated with higher perioperative mortality and a substantial risk of graft failure may not be justified. Additionally, transplantation in high-risk patients may preclude organ allocation for other candidates with a higher likelihood of survival. Nevertheless, in some cases, attempting transplantation may represent the only potential opportunity to alter an otherwise poor prognosis.

The heterogeneity in anatomical and pathological findings in this population, particularly related to the pulmonary arteries and prior surgical interventions, underscores the importance of tailoring the surgical strategy to the specific needs of each case. Notably, none of the patients with single-ventricle physiology underwent heart transplantation due to unoperated hypoplastic left heart syndrome. The increasing success of palliative interventions has allowed some of these patients to survive into adulthood. However, a consequence of this success is the complex surgical approach required for patients undergoing transplantation due to palliative treatment failure. Consistent with data from other centers, the primary indication for transplantation in our population with single-ventricle hearts was failing Fontan physiology [24].

Optimal pulmonary artery reconstruction remained a critical factor in the success of heart transplantation in this cohort. Harvesting the donor heart with pulmonary arteries extending to the hilar level was essential to facilitate effective reconstruction. In cases of multiorgan procurement in which the lungs are also harvested, careful pre-procurement planning for pulmonary trunk reconstruction is imperative. The use of a Dacron prosthesis in one patient was technically challenging due to its synthetic structure, which complicated the sizing and implantation process. The application of a homograft in such situations could potentially offer greater benefits [25,26].

Uncontrolled bleeding was a recurring issue during surgery, often exacerbated by coagulopathy resulting from prior cavopulmonary connections and congestive liver dysfunction. Effective hemostatic management, including advanced surgical techniques and perioperative coagulation support, was essential in managing this complication. Another significant challenge was the persistence of protein-losing enteropathy in some patients, a common sequela of failing Fontan physiology. Although protein-losing enteropathy persisted in the immediate postoperative period in several cases, it resolved in all patients who survived the perioperative phase. This finding emphasizes the transformative impact of successful transplantation on systemic complications associated with Fontan physiology, even in severely debilitated patients.

## 5. Limitations

This study is limited by its small, single-center, retrospective design, reflecting the rarity of heart transplantation in patients with single-ventricle physiology. The heterogeneity of the cohort, the long study period, and evolving surgical and perioperative practices may affect the generalizability of our findings. The low event rate and extended study period further limit generalizability and preclude robust survival analyses.

## 6. Conclusions

Our findings reinforce the necessity of a multidisciplinary approach to heart transplantation in single-ventricle patients. Surgeons must anticipate the unique anatomical challenges, including pulmonary artery abnormalities and prior surgical adhesions, and plan accordingly [22,27]. Additionally, careful consideration should be given to the type of graft material used for pulmonary artery reconstruction. Future efforts should focus on refining surgical techniques, improving perioperative care, and exploring novel graft materials to optimize outcomes for this challenging patient population.

## Figures and Tables

**Table 1:** Clinical Characteristics of Patients Undergoing Heart Transplantation.

Patient No.	Age/Sex/Weight (kg)	Preexisting Diagnoses	Imaging & Hemodynamics	Indication & Functional Status	Baseline Labs	Cannulation & Ischemic Time	CPB Time & Vascular Reconstruction	Early Postoperative Complications
1	20/F/69	Heterotaxy syndrome; DOLV; hypoplastic RV; common atrium; hypoplastic LPA; status post bidirectional Glenn; PA occlusion. TIA; venous sinus thrombosis; recurrent DVT; chronic venous insufficiency; gout.	Mild-moderate MR; pulmonary stenosis; reduced LV systolic function; PH: no (Fontan).	Increasing cyanosis; recurrent thrombosis; malignant arrhythmias; NYHA III-IV; CPET peak VO_2_ 7.4 mL/kg/min (18% predicted).	NT-proBNP 436.3 pg/mL; protein 62 g/L; albumin 37 g/L; bilirubin 6.7 µmol/L; creatinine 78 µmol/L.	Aorta (20 EOPA) + femoral artery + SVC + IVC; ischemic time 194 min.	CPB 292 min; PA reconstruction with donor graft.	Secondary anemia; left brachial vein thrombosis; CMV infection; PTSD.
2	7/F/15	HLHS; Norwood; Hemi-Fontan; recurrent aortic coarctation treated with balloon angioplasty; LPA stenting.	Good ventricular contractility; severe systemic valvular insufficiency; AI I-II; ReCoA gradient 24 mmHg; PAP 23/20/21 mmHg; McGoon index 2.1; PVR 2.82 WU.	NYHA IV.	Protein 77 g/L; bilirubin 8.4 µmol/L; creatinine 60 µmol/L; PRA 22.6%.	Aorta + SVC + IVC; ischemic time 374 min.	CPB 260 min; PA reconstruction with Dacron graft.	Severe HF; re-exploration for bleeding; open chest; VA-ECMO → VV-ECMO; renal failure (HDF); bloodstream infection; acute rejection; tracheostomy; PEG; pneumothorax; death.
3	31/F/61	Tricuspid atresia; VSD; pulmonary stenosis; dextroversion; bilateral modified Blalock–Taussig shunt; Fontan (lateral tunnel); LPA stenting; MAPCA closures.	Severe mitral regurgitation; massively dilated lateral tunnel; INTERMACS 3.	NYHA IV; ascites; impaired liver function; varices.	data not available	Femoral artery + SVC + IVC; ischemic time 214 min.	CPB 114 min; PA reconstruction with donor graft.	Intraoperative bleeding; reoperation for bleeding; IABP; acute renal failure (HDF).
4	14/F/27	HLHS; right ventricular anomaly; bidirectional Glenn.	LV EF 45%; moderate MR; dilated hepatic veins; free abdominal fluid; CO 3.6 L/min.	End-stage HF (failing Glenn).	NT-proBNP 3850 pg/mL; protein 39 g/L; albumin 23 g/L; bilirubin 5.6 µmol/L; PRA 10%.	Aorta + bicaval; ischemic time 186 min.	PA reconstruction with donor graft.	None.
5	24/M/57	Tricuspid atresia; pulmonary atresia; Blalock–Taussig shunt; Bjork–Fontan.	Single-ventricle EF 12%.	RV failure; increasing cyanosis; protein-losing enteropathy.	data not available	Aorta + bicaval; ischemic time 136 min.	CPB 105 min; PA reconstruction with pericardial patch.	None.
6	18/M/47.9	Tricuspid atresia; TGA; VSD; PA banding; Fontan; IVC-RA stent; infective endocarditis; left kidney agenesis.	data not available	NYHA IV; ascites; protein-losing enteropathy.	Protein 67 g/L; albumin 45 g/L; bilirubin 16.9 µmol/L; creatinine 70 µmol/L; PRA 50%.	Right femoral artery and vein; right internal jugular vein; ischemic time 274 min.	CPB 240 min; PA reconstruction with donor graft.	Subdiaphragmatic abscess; fenestration via minilaparotomy.
7	15/M/41	HLHS; Norwood; hemi-Fontan; Fontan with fenestration; LPA stenting; epilepsy; Schönlein–Henoch purpura; hypothyroidism.	Systemic RV failure; moderate TR; mild AI.	NYHA IV; INTERMACS 3; catecholamine-dependent; ventricular arrhythmias.	PRA 0%.	Right femoral artery and vein; right internal jugular vein; ischemic time 247 min.	CPB 134 min; PA reconstruction with pericardial patch.	None.
8	17/M/51.1	HLHS; Norwood; hemi-Fontan; MAPCA occlusion; LIMA/RIMA occlusion.	Systemic RV EF 30%; severe TR; moderate neoaortic regurgitation; large ASD.	End-stage HF (failing Fontan).	NT-proBNP 2878 pg/mL; protein 83 g/L; albumin 43 g/L; bilirubin 35.6 µmol/L; PRA 0%.	Aorta + SVC + RA; ischemic time 217 min.	CPB 178 min; PA reconstruction with donor graft.	None.
9	9/F/23	Tricuspid atresia; pulmonary stenosis; VSD; Damman-Miller; Fontan.	Reduced ventricular function; moderate mitral insufficiency.	End-stage HF (failing Fontan).	data not available	Aorta + SVC + RA; ischemic time 185 min.	CPB 160 min; PA reconstruction with autologous aortic patch to RPA.	Pulmonary cytomegalovirus infection (cause of death).

Abbreviations: AI—aortic insufficiency, ASD—atrial septum defect, CPB—cardiopulmonary bypass, CPET—Cardiopulmonary exercise testing, EF—ejection fraction, EOPA—elongated one-piece aortic cannula, F—female, HDF—hemodiafiltration, HF—heart failure, HLHS—Hypoplastic left heart syndrome, iNO—inhaled nitric oxide, IVC—inferior vena cava, LPA—left pulmonary artery, M—male, PAP—pulmonary artery pressure, PH—pulmonary hypertension, PA—PS—pulmonary stenosis, PVR—pulmonary vascular resistance, RA—right atrium, ReCoA—recurrent aortic coarctation, RPA—right pulmonary artery RV—right ventricle, SVC—superior vena cava, TA—tricuspid atresia, TGA—Transposition of the Great Arteries, VA—veno-arterial, VSD—ventricular septal defect, VV—veno-venous, Tx—transplantation.

## Data Availability

Additional clinical data will be made available by the corresponding author in clinically justified cases and in accordance with the protection of patient personal data.

## Abbreviations

The following abbreviations are used in this manuscript:

CHD	congenital heart disease
F	female
HF	heart failure
HLHS	hypoplastic left heart syndrome
ISHLT	International Society for Heart and Lung Transplantation
LIMA	left interior mammary artery
M	male
MAPCA	major aortopulmonary collateral arteries
OHT	orthotopic heart transplantation
RIMA	right interior mammary artery
PLE	protein-losing enteropathy
